# A structured approach to address variability in the global management of adrenal insufficiency in Duchenne muscular dystrophy: the 2025 PJ Nicholoff steroid protocol

**DOI:** 10.3389/fneur.2026.1926817

**Published:** 2026-09-10

**Authors:** Anne Marie Sbrocchi, Alexandra Ahmet, Kathi Kinnett, Maria-Elena Lautatzis, Hugh J. McMillan, Susan Apkon, Meilan M. Rutter, Leanne M. Ward, Sasigarn A. Bowden, Anne M. Connolly, Garey Noritz, Rachel Schrader, Kathryn Selby, Aravindhan Veerapandiyan, Sze Choong Wong, David R. Weber

**Affiliations:** 1 Division of Pediatrics and Metabolism, Montreal Children’s Hospital, McGill University Health Center, Montreal, QC, Canada; 2 Division of Endocrinology and Metabolism, Department of Pediatrics, Children’s Hospital of Eastern Ontario, University of Ottawa, Ottawa, ON, Canada; 3 Parent Project Muscular Dystrophy (PPMD), Washington, DC, United States; 4 Department of Pediatrics and Child Health, Children's Hospital of Manitoba, University of Manitoba, Winnipeg, MB, Canada; 5 Department of Physical Medicine and Rehabilitation, Children's Hospital Colorado and Anschutz University of Colorado School of Medicine, Aurora, CO, United States; 6 Division of Diabetes and Endocrinology, Cincinnati Children’s Hospital Medical Center, University of Cincinnati College of Medicine, Cincinnati, OH, United States; 7 Department of Pediatrics, Nationwide Children’s Hospital, Ohio State University, Columbus, OH, United States; 8 Division of Neurology, Department of Pediatrics, British Columbia Children's Hospital, University of British Columbia, Vancouver, BC, Canada; 9 Department of Pediatrics, Arkansas Children's Hospital, University of Arkansas for Medical Sciences, Little Rock, AR, United States; 10 Bone, Endocrine, Nutrition Research Group in Glasgow, University of Glasgow, Glasgow, United Kingdom; 11 Department of Pediatric Endocrinology, Royal Hospital for Children, University of Glasgow, Glasgow, United Kingdom; 12 Division of Pediatric Endocrinology and Diabetes, The Children’s Hospital of Philadelphia and the Perelman School of Medicine at the University of Pennsylvania, Philadelphia, PA, United States

**Keywords:** adrenal insufficiency, corticosteriods, duchenne, glucocorticoid, vamorolone

## Abstract

Adrenal suppression is a potentially life-threatening consequence of chronic glucocorticoid (GC) therapy in Duchenne muscular dystrophy (DMD), placing individuals at risk for adrenal insufficiency and adrenal crisis during increased physiological stress, missed GC doses, abrupt discontinuation, or transitions between GC regimens. Management is increasingly complex because DMD treatment now includes multiple GC agents, including prednisone/prednisolone, deflazacort, and vamorolone, as well as daily, intermittent, and transition regimens that differ across countries and clinical settings. Although general principles of adrenal insufficiency prevention are well established, implementation remains challenging because of global variability in drug availability, institutional stress-steroid protocols, clinician experience, and caregiver education. This article discusses key sources of variability in adrenal suppression management for individuals with DMD and presents the 2025 PJ Nicholoff Steroid Protocol as an example of a practical, DMD-specific framework. The updated protocol incorporates newer therapies such as vamorolone and emphasizes anticipatory education, written stress-steroid plans (including a weight-based approach), safe GC tapering, confirmation of hypothalamic–pituitary–adrenal axis recovery, and careful management of transitions between GC agents and regimens. This structured approach aims to support safer and more consistent adrenal suppression management for individuals with DMD receiving chronic GC therapy through six critical concepts.

## 1 Introduction

Adrenal suppression is a complication of chronic glucocorticoid (GC) therapy used to treat people with Duchenne muscular dystrophy (DMD), resulting in decreased endogenous cortisol production from suppression of the hypothalamic–pituitary–adrenal axis (HPA) axis. During increased physiologic stress, treatment doses may be insufficient, resulting in symptomatic adrenal insufficiency. If additional GCs (“stress-steroids”) are not given during times of physiological stress, these individuals are at risk of an adrenal crisis manifesting with hypotension and electrolyte abnormalities (1). They are also at risk if GCs doses are missed or abruptly discontinued. Risk may be heightened during transition periods, including changes between GC regimens. Terminology describing the pathophysiology and clinical manifestations of adrenal suppression is often inconsistent and imprecise. Throughout this manuscript, *HPA axis suppression* refers to reduced endogenous cortisol production caused by exogenous GC exposure; *adrenal insufficiency* refers to inadequate cortisol availability for basal or stress needs; *maintenance* (also referred to as physiologic) replacement refers to GC dosing intended to approximate normal cortisol production (approximately 8–10 mg/m^2^/day of exogeneous hydrocortisone); and *triple-maintenance* refers to an approximate supraphysiologic dose equal to three times physiologic replacement used for stress steroid dosing.

All GCs used to treat DMD (prednisone, prednisolone, deflazacort and recently approved vamorolone) suppress the HPA axis when administered daily at standard DMD treatment doses (2, 3). Prednisone, prednisolone, and deflazacort are classic GCs that signal through the GC receptor, exerting effects through both transrepression and transactivation (4). GC-receptor transrepression mediates anti-inflammatory benefit by suppressing pro-inflammatory pathways, whereas transactivation drives adverse effects through direct gene activation. Importantly, adrenal suppression is a result of transrepression (5). Vamorolone selectively modulates GC receptor activity, dissociating transrepression from transactivation, preserving the beneficial anti-inflammatory effects resulting from transrepression, while reducing some adverse effects of transactivation (6). Vamorolone is also a mineralocorticoid receptor (MR) antagonist, unlike classic GCs, which have neutral or agonist MR effects (7). This MR antagonism further complicates adrenal suppression management during increased physiologic stress, possibly increasing the risk for hypotension and electrolyte abnormalities when MR signaling plays a critical role in supporting blood pressure (8).

This manuscript discusses sources of global variability in adrenal suppression management for GC- treated individuals with DMD and provides updated considerations that include vamorolone. It presents the revised PJ Nicholoff Steroid Protocol, as an example of a structured, DMD-specific framework for managing adrenal suppression (9). The protocol was originally published in 2017 following the death of PJ Nicholoff, a young man with DMD who did not receive his prescribed GCs or stress-dose steroids in hospital. These guidelines are also applicable to individuals with related dystrophinopathies treated with GCs (Becker muscular dystrophy, female manifesting carriers of DMD). Although GC use in these conditions is off label and not standard of care, they may be prescribed for severely affected individuals, resulting in the same risks of adrenal suppression as occur in people with DMD treated with GCs.

### 1.1 Global variability in clinical treatment of DMD

GC regimens vary worldwide based on specific drug availability, labeling, reimbursement, and individual/clinician preference. Prednisone, prednisolone, deflazacort, and vamorolone demonstrate efficacy in slowing disease progression and preserving muscle strength (10–14). GC selection and dosing regimen are individualized to balance therapeutic benefit against adverse effects. Daily prednisone/prednisolone (0.75 mg/kg, maximum dose 30–40 mg), deflazacort (0.9 mg/kg, maximum dose 36 mg), and vamorolone (6 mg/kg; maximum dose 300 mg in the United States, and 240 mg in the EU, United Kingdom, China, and Canada) are commonly used first-line regimens, while twice-weekly prednisone or deflazacort and other reduced or intermittent dosing strategies are used in some centers to improve tolerability. Vamorolone showed comparable efficacy on functional outcomes to daily prednisone with less growth and bone turnover suppression (12–14), however adrenal suppression and excess weight gain still occurred (2, 14).

### 1.2 Challenges and controversies in managing adrenal suppression

Variability in adrenal suppression management is multifactorial, reflecting differences in GC properties, stress-dosing and tapering practices, and healthcare infrastructure, including access to specific GC formulations and experienced clinicians.

Definitions of “moderate” and “severe” physiologic stress vary and contribute to differences in prescribed stress steroid regimens. Although hydrocortisone is the standard agent for stress dosing (15, 16), some clinicians instruct families to double or divide their regular DMD GC treatment dose, but this strategy has not been studied. This approach may be appropriate for individuals taking classic GCs (prednisone, prednisolone, deflazacort) but is complicated by differences in their pharmacokinetic properties. Prednisone/prednisolone have longer half-lives such that twice-daily dosing should provide adequate stress coverage, whereas deflazacort lacks MR agonism and has a shorter half-life, potentially requiring more frequent dosing for effective stress coverage (3, 17). Importantly, vamorolone is not appropriate for stress coverage because its MR antagonism may compromise blood pressure regulation during physiologic stress Mechanistic studies have confirmed the MR antagonistic properties of vamorolone (2, 7), and clinical evidence of MR antagonism in vamorolone treated individuals has been reported (8).

Another unresolved issue is whether individuals on twice-weekly or other intermittent GC regimens should be presumed to have adrenal suppression and require stress-steroid dosing. Some experts advocate universal stress-dosing education and intramuscular (IM) hydrocortisone preparedness for all such individuals, concerned that intermittent dosing may attenuate HPA axis responsiveness (18). Others suggest intermittent regimens may allow HPA axis recovery and support an individualized approach.

Approaches to GC tapering and confirmation of HPA axis recovery after GC discontinuation also remain controversial. No universally accepted standards exist for tapering rate, duration, or criteria for adrenal recovery. Some centers use multi-step tapering protocols with biochemical confirmation of HPA axis recovery before discontinuing physiologic replacement and stress coverage, while others individualize tapering based on clinical tolerance. Methods to assess HPA axis recovery also vary, with some clinicians relying on morning cortisol screening and others using adrenocorticotropic hormone (ACTH) stimulation testing (2, 19).

Together, these clinical, pharmacologic, and logistical challenges result in substantial variability in adrenal suppression management among individuals with DMD treated with GCs. The introduction of vamorolone has further highlighted the limitations of existing practices. Although existing clinical frameworks provide guidance (16, 19, 20), they are not tailored to the unique and evolving therapeutic complexities of people with DMD treated with GCs.

### 1.3 The revised PJ Nicholoff steroid protocol (2025): a framework for the management of adrenal suppression in people with DMD

The PJ Nicholoff Steroid Protocol was first published to educate clinicians caring for people with DMD about adrenal suppression and its management (21). Subsequent therapeutic advances, including vamorolone, have introduced new challenges.

The 2025 update was developed through regular meetings of the OPTIMIZE DMD Adrenal Insufficiency Working Group and broader consortium contributors, iterative review of the protocol and manuscript, and targeted review of the relevant DMD and adrenal insufficiency literature. Recommendations directly supported by published clinical studies or established adrenal insufficiency guidelines are identified as evidence-informed; recommendations addressing DMD-specific situations for which direct data are lacking are identified as expert consensus. This approach was selected to provide practical guidance while transparently acknowledging areas of uncertainty. The expanded edition aims to improve consistency and safety across the DMD community, emphasizing early recognition, anticipatory education, and structured management through six critical concepts.

### 1.4 Critical concepts

#### 1.4.1 Individuals with DMD taking daily supraphysiologic GC doses are at risk for GC induced adrenal suppression and adrenal insufficiency

At baseline, corticotropin-releasing hormone stimulates ACTH secretion, resulting in cortisol production (maintenance), which may increase several-fold more during physiologic stress (15, 16, 22).

All daily GC regimens (prednisone/prednisolone, deflazacort, vamorolone) exceed physiologic cortisol production and suppress the HPA axis, with risk of clinically significant adrenal suppression after 2 to 4 weeks (21, 23) leading to impairment of the stress response (15). Without timely stress dosing, adrenal insufficiency may progress to adrenal crisis with hypotension, hypoglycemia, altered consciousness, or death (1). Physiologic (maintenance) doses for the different GCs vary according to potency and are only known for prednisone/prednisolone; weight-based maintenance equivalent comparisons may also be derived from body surface area (BSA) (24). Although individuals on twice-weekly or other intermittent GC regimens are likely at lower risk of adrenal suppression they may potentially develop symptomatic adrenal insufficiency, particularly in the day or two following exposure, however comprehensive data are lacking.

#### 1.4.2 Signs and symptoms of adrenal insufficiency are often nonspecific and may only develop when the individual is under increased physiologic stress

Individuals and caregivers should be counseled on the signs and symptoms of adrenal insufficiency (Supplementary Table 1) before or at GC initiation, with reinforcement at least annually, and instructed to never stop or withhold GC doses (23). Symptoms of GC-induced adrenal insufficiency may include anorexia, nausea/vomiting, malaise, weakness or fatigue, headache, abdominal pain, myalgia/arthralgia, psychiatric symptoms (confusion, delirium, disorientation) and loss of motor skills (out of keeping with natural progression). Signs of GC-induced adrenal insufficiency include hypotension, hypoglycemia, tachycardia and bradycardia. Fever is an uncommon sign of adrenal insufficiency but may occur in isolation in rare instances. The source of fever should always be investigated and treated, as appropriate. GC withdrawal is a distinct, non-life-threatening entity that occurs when tapering GCs too rapidly while on supraphysiologic doses, yet its symptoms resemble adrenal insufficiency (19). Hypotension and hypoglycemia typically do not occur with GC withdrawal.

#### 1.4.3 All individuals on chronic daily supraphysiologic doses of GCs must have a stress-steroid plan

Additional (“stress”) steroids are required during periods of increased physiologic stress (illness, injury, surgery), to prevent adrenal crisis (Supplementary Table 2) (23, 25). Hydrocortisone, identical to endogenous cortisol, is the ideal choice for stress coverage. Prednisone/prednisolone, which are more potent and longer acting, are appropriate alternatives for moderate stress (17). Vamorolone cannot be used for stress dosing because its MR antagonism may worsen hypotension and electrolyte disturbances during physiologic stress, especially if there is concurrent use of renin-angiotensin-aldosterone system inhibitors (e.g., spironolactone, eplerenone) (2, 7, 8). There are insufficient data to recommend deflazacort for stress dosing and there are concerns regarding its short half-life and limited MR agonism (16). Stress-steroids are typically administered in addition to the DMD treatment GC, except for those taking prednisone/prednisolone, in whom the daily treatment dose may be equally divided and given twice-daily. There is no consensus as to whether or not Individuals on twice-weekly or other intermittent GC regimens should have a formal stress steroid plan, however they must be educated about the possibility of adrenal insufficiency. Individuals transitioning from a daily to twice-weekly regimen must continue a stress steroid plan until the HPA axis recovers.

**Figure 1:**
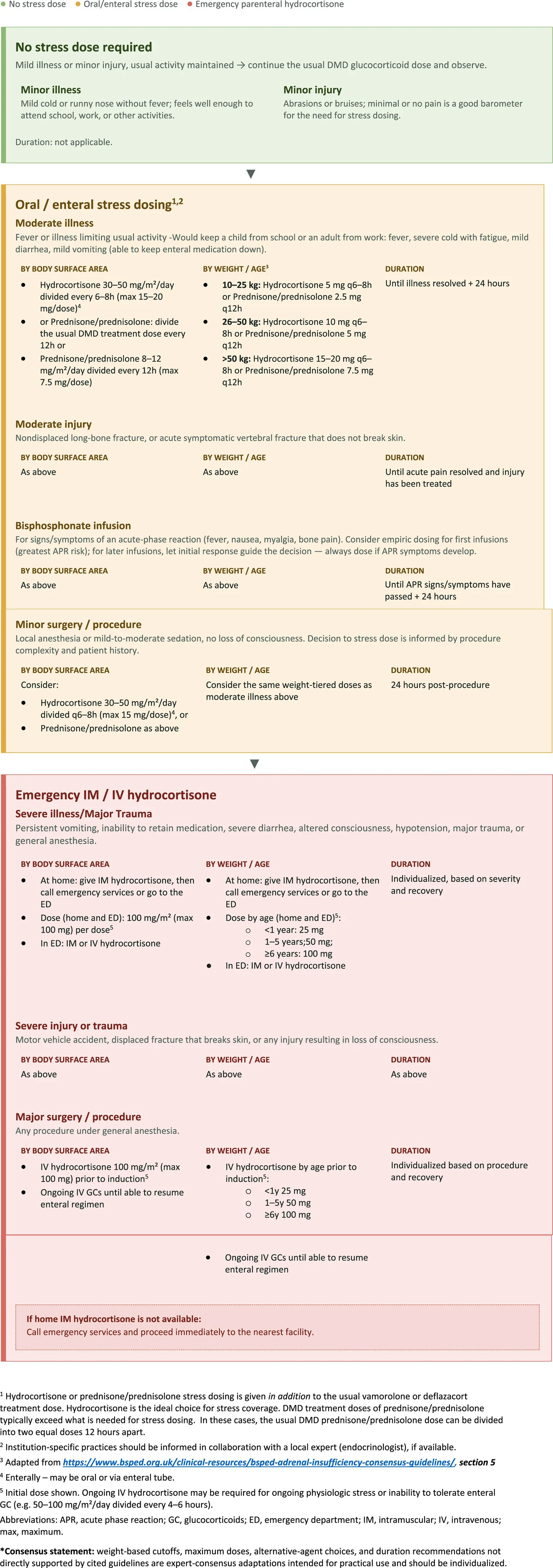
Indications, doses and examples for moderate and severe stress steroid dosing.

Stress severity (moderate, or severe) determines the route, frequency, and dose of stress steroids (Figure 1). Moderate stress-dosing with hydrocortisone or prednisone/prednisolone may be based on BSA (m^2^) or weight (kg) (19, 20). IM or intravenous (IV) hydrocortisone is required for severe physiologic stress or when enteral dosing is not tolerated (BSA or age-based dose); continuous IV hydrocortisone infusions for severe stress may be used (Supplementary Table 3) (20). Individuals should have medical identification/accessories indicating steroid dependence, always have their stress-dosing plan available, and have adrenal insufficiency flagged in the medical record (26).

#### 1.4.4 A GC taper must be implemented when decreasing or discontinuing GCs in individuals who have been treated with daily supraphysiologic doses for greater than 2-to-4 weeks

**Figure 2:**
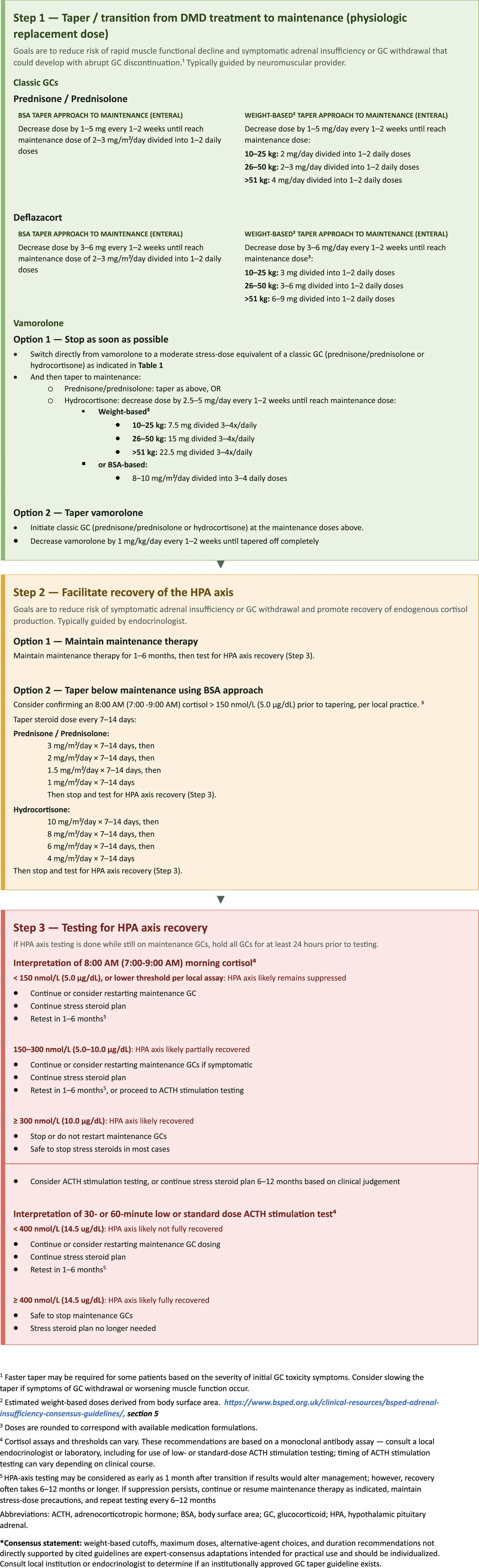
Example approaches to tapering/transitioning off Glucocorticoids (GCs) and testing for Hypothalamic–pituitary–adrenal axis (HPA axis) recovery.

Abrupt GC cessation increases the risk of adrenal crisis and GC withdrawal. A structured taper with careful monitoring supports safe reduction and HPA axis recovery while minimizing withdrawal symptoms (19). There is insufficient evidence to support a single tapering strategy and gradual tapering does not guarantee adrenal crisis prevention (23). An example approach to tapering different GCs is provided (Figure 2) for use when local protocols are unavailable. The tapering intervals and weight-based doses are expert-opinion examples derived from physiologic replacement calculations (24), available formulations, and consortium experience. The example protocol includes three steps: (1) Taper/transition from DMD treatment dose to physiological dose (maintenance), (2) Facilitate HPA axis recovery, (3) Assess HPA axis recovery and discontinue maintenance and stress-steroid plans once recovery confirmed (27).

#### 1.4.5 Individuals tapering off daily GCs or on physiologic (maintenance) doses must continue to take stress-steroids during periods of increased physiologic stress until recovery of the HPA axis has been confirmed

Stress-steroid plans must continue to be followed until HPA axis recovery has been confirmed by morning cortisol and/or ACTH stimulation testing, depending on local practices (Figure 2). With long-term GC therapy, HPA axis recovery may take 6–12 months or longer (19).

#### 1.4.6 When transitioning from one GC regimen to another, care must be taken to reduce the risk of adrenal insufficiency or GC withdrawal

Transitions between GCs require direction by an experienced DMD healthcare provider with endocrinology consultation, when feasible, to reduce the risk of symptomatic adrenal insufficiency including adrenal crisis or GC withdrawal symptoms (Supplementary Table 4). The suggested transition approaches reflect expert consensus and have not been formally evaluated.

##### 1.4.6.1 Transitioning from one daily to another daily GC

When transitioning from daily prednisone/prednisolone or deflazacort to vamorolone at 6 mg/kg/day or the maximum approved dose (300 mg in the USA, 240 mg in the EU, UK, China, and Canada), tapering is not required and individuals can switch directly to the new regimen (recommended approach). Similarly, when transitioning from vamorolone to prednisone/prednisolone 0.75 mg/kg/day or deflazacort 0.9 mg/kg/day, or the maximum dose determined by the prescribing clinician, a direct switch can be made without tapering. The first day of the new GC dosing must directly follow the last day of the previous GC such that no doses are missed.

Additional GC coverage may be required when transitioning to lower-dose regimens or if GC withdrawal symptoms develop when transitioning to standard dose regimens. For individuals switching from daily prednisone/prednisolone or deflazacort to vamorolone at a dose below 6 mg/kg/day, or below the maximum approved dose, hydrocortisone or prednisone/prednisolone should be initiated at a maintenance dose (Figure 2, Step 1) in addition to the desired vamorolone dose. This maintenance coverage can be continued for approximately 2–4 weeks after the transition to facilitate a smooth change between therapies and then discontinued if the individual remains clinically stable. If symptoms of GC withdrawal develop, stress dosing should be initiated according to the individual’s stress-steroid plan, followed by a gradual taper back to maintenance dosing. When transitioning from vamorolone to prednisone/prednisolone doses below 0.75 mg/kg/day or deflazacort doses below 0.9 mg/kg/day, the approach depends on whether the target dose provides adequate supraphysiologic GC coverage. A direct switch without tapering may be appropriate only if the desired prednisone/prednisolone or deflazacort dose exceeds the following threshold doses (approximately equivalent to triple-maintenance), rounded to correspond with available medication formulations. For prednisone/prednisolone these thresholds are approximately 5 mg daily for individuals weighing 10–25 kg, 10 mg daily for those weighing 26–50 kg, and 15 mg daily for those weighing over 51 kg. For deflazacort, these thresholds are approximately 6 mg daily for individuals weighing 10–25 kg, 12 mg daily for those weighing 26–50 kg, and 18 mg daily for those weighing over 51 kg. These weight brackets and triple-maintenance thresholds are derived from BSA-based physiologic replacement estimates (24) and available medication formulations. If the desired dose is below these thresholds, the individual should first be switched to a prednisone/prednisolone or deflazacort dose that provides supraphysiologic coverage, followed by gradual tapering by 2.5–5 mg/day for prednisone/prednisolone or 3–6 mg/day for deflazacort every 1–2 weeks until the desired dose is reached.

##### 1.4.6.2 Transitioning from daily GC to twice-weekly or other intermittent GC regimens

Alternative GC regimens including twice-weekly or 10 days on 10 days off are sometimes used to reduce the impact of GC related side effects, though the 10 days on 10 days off regimen is inferior for motor function and the twice-weekly one requires longer study (28, 29). Although the adrenal suppression risk of twice-weekly or intermittent regimens is likely less than for daily dosing, it may occur during transitions from daily to twice-weekly or intermittent dosing, particularly on “off days” (days GCs not administered). Stress dosing should be provided as needed throughout any transition.

Because direct evidence is insufficient and no consensus currently exists, this framework recommends a precautionary default: individuals transitioning from daily therapy, those with symptoms suggestive of adrenal insufficiency, or those whose HPA axis status is unknown should be managed as potentially suppressed and provided with stress-steroid education and a written plan until recovery is demonstrated. For individuals established on an intermittent regimen with documented HPA axis recovery, routine stress dosing is not required.

##### 1.4.6.3 Twice-weekly GC regimen transitions

Transitions from daily prednisone/prednisolone, deflazacort, or vamorolone to twice-weekly prednisone/prednisolone or deflazacort should generally occur over approximately 6–8 weeks. During this transition, GC doses on “off days” should be gradually reduced while equivalent doses are increased on treatment days until the twice-weekly regimen is reached. If symptoms of GC withdrawal or adrenal insufficiency develop on “off days,” the previous “off-day” dose should be restarted and the “off-day” taper should proceed more gradually, in parallel with increases on treatment days.

##### 1.4.6.4 Other intermittent GC regimen transitions

For transitions from daily prednisone/prednisolone, deflazacort, or vamorolone to intermittent prednisone/prednisolone or deflazacort regimens other than twice-weekly dosing, such as 10 days on and 10 days off, additional “off-day” coverage should be considered. On “off days,” maintenance prednisone/prednisolone or hydrocortisone can be started (Figure 2, Step 1) and continued for approximately 4 weeks before stopping if the individual remains clinically stable. If symptoms of GC withdrawal develop, maintenance dosing should be restarted, and a longer duration of treatment or a taper off maintenance doses should be considered.

On “treatment days” during intermittent regimens, the approach depends on whether the intermittent prednisone/prednisolone or deflazacort dose exceeds supraphysiologic, or triple-maintenance, thresholds as indicated above. If the “treatment-day” dose exceeds these thresholds, the individual can switch directly to the desired intermittent “treatment-day” dose. If the desired “treatment-day” dose is below maintenance, the individual should first receive a supraphysiologic prednisone/prednisolone or deflazacort dose on “treatment days,” followed by gradual tapering by 2.5–5 mg/day for prednisone/prednisolone or 3–6 mg/day for deflazacort every 1–2 weeks until the desired intermittent “treatment-day” dose is reached.

Endocrinology consultation is recommended when available. HPA-axis testing may be considered as early as 1 month after transition when the result would change immediate management; however, early testing is expected to remain abnormal in many individuals because overall recovery commonly takes 6–12 months or longer. If an early morning cortisol or ACTH stimulation test indicates persistent suppression, maintenance therapy should be continued or restarted when clinically indicated, the stress-steroid plan should remain active, and testing should generally be repeated every 6–12 months, with shorter intervals reserved for situations in which the result would alter treatment. (Figure 2, Step 3). Vamorolone should not be used for twice-weekly or intermittent regimens due to its pharmacologic profile and lack of evidence supporting safe intermittent use.

### 1.5 Minimum-standard approach

Where endocrinology consultation, IM hydrocortisone, or cortisol testing is not readily available, the minimum standard is to: (1) never abruptly stop chronic daily GCs; (2) provide a written plan using available enteral hydrocortisone or prednisone/prednisolone for moderate illness; (3) direct patients with persistent vomiting, altered consciousness, hypotension, severe injury, or inability to retain enteral medication to urgent medical care for parenteral hydrocortisone; (4) continue stress-steroid precautions after tapering or transition until recovery can be assessed or for a minimum of 12 months; and (5) document steroid dependence prominently in the medical record and use medical identification.

## 2 Discussion

The expanding range of GC regimens for the treatment of DMD allows greater individualization of therapy based on clinical response and tolerability. However, this diversity complicates adrenal suppression management, limiting the feasibility of a universal stress-dosing strategy and standardized patient/caregiver education applicable across all GC agents and regimens. Global differences in healthcare infrastructure also influence implementation of stress steroid protocols and emergency preparedness. Families are often provided with written stress-dosing plans, medical identification bracelets, and IM hydrocortisone for home use; however, instructions may be complex, emergency kits inconsistently supplied or available, and caregivers and physicians may lack confidence in recognizing or managing adrenal crises (30–33). Comparable quantitative international data on regimen use, emergency-kit access, and adrenal-crisis outcomes remain limited. Accordingly, statements regarding global variability should be interpreted as reflecting documented differences in practice and infrastructure rather than a precise estimate of prevalence.

The revised 2025 PJ Nicholoff Steroid Protocol provides a practical framework to support safer and more consistent adrenal suppression management for individuals with DMD receiving various chronic GC therapeutic regimens. In principle, the management of adrenal insufficiency by avoiding missed doses, tapering GCs when discontinuing therapy, and administering extra GC doses during physiologic stress is straightforward and guidelines exist (16, 19, 20). In practice, implementation is challenging due to the global variability in the GC agents and regimens used for DMD, limited access to clinicians experienced in adrenal suppression, institutional differences in stress-steroid and GC tapering protocols, and the relative infrequency of symptomatic adrenal insufficiency and adrenal crisis. These barriers may contribute to survey findings of limited awareness and discomfort with adrenal insufficiency management among clinicians and caregivers (31–33). This structured approach aims to facilitate removal of these barriers by supporting safer and more consistent adrenal suppression management for individuals with DMD receiving chronic GC therapy, while recognizing that clinical practices differ due to lack of evidence.

## 3 Limitations

This protocol was developed through iterative expert consensus and targeted literature review rather than a formal Delphi process, systematic review, or graded evidence framework. Direct DMD-specific evidence is limited for stress-dosing alternatives, intermittent regimens, transition coverage, tapering intervals, weight-based thresholds, and the timing of HPA-axis recovery testing. These recommendations should therefore be individualized, updated as new evidence emerges, and evaluated prospectively.

## 4 Conclusion

No universal, evidence-based protocol exists for adrenal suppression management in individuals with DMD treated with GCs. The updated 2025 PJ Nicholoff Steroid Protocol provides an example of a structured framework through six critical concepts that can be implemented globally to ensure knowledge and protection from adrenal crisis in the evolving era of GC therapy.

## Data Availability

The original contributions presented in the study are included in the article/Supplementary material, further inquiries can be directed to the corresponding authors.

## Group members of on behalf of the OPTIMIZE DMD Consortium

Steering Committee: Chair - Leanne M. Ward (Children’s Hospital of Eastern Ontario, University of Ottawa, Ottawa, Ontario, Canada); Co-Chairs - Pat Furlong (Parent Project Muscular Dystrophy), David R. Weber (The Children’s Hospital of Philadelphia, University of Pennsylvania, Philadelphia, PA, United States), and Sze Choong Wong (Royal Hospital for Children, University of Glasgow, Glasgow, United Kingdom); Members - Hugh J. McMillan (Children’s Hospital of Eastern Ontario, University of Ottawa, Ottawa, Ontario, Canada), Meilan M. Rutter (Cincinnati Children’s Hospital Medical Center, University of Cincinnati College of Medicine, Cincinnati, Ohio, United States), Susan Apkon (Children’s Hospital Colorado, University of Colorado School of Medicine, Aurora, CO, United States), and Jaclyn Tamaroff (Vanderbilt University Medical Center, Nashville, TN, United States). OPTIMIZE DMD Headquarters Personnel - Roozbeh Pourziaei Manesh and Mei Wang (The Ottawa Pediatric Bone Health Research Group, Children’s Hospital of Eastern Ontario Research Institute, Ottawa, Ontario, Canada). Adrenal Insufficiency Working Group: Co-Chairs - Anne Marie Sbrocchi (Montreal Children’s Hospital, McGill University, Montreal, Québec, Canada) and David R. Weber (The Children’s Hospital of Philadelphia, University of Pennsylvania, Philadelphia, PA, United States); Members - Alexandra Ahmet (Children’s Hospital of Eastern Ontario, University of Ottawa, Ottawa, Ontario, Canada), Aravindhan Veerapandiyan (Arkansas Children’s Hospital, University of Arkansas for Medical Sciences, Little Rock, AR, United States), Hugh J. McMillan (Children’s Hospital of Eastern Ontario, University of Ottawa, Ottawa, Ontario, Canada), Sze Choong Wong (Royal Hospital for Children, University of Glasgow, Glasgow, United Kingdom), Kathryn Selby (British Columbia Children’s Hospital, The University of British Columbia, Vancouver, British Columbia, Canada), Maria-Elena Lautatzis (Manitoba Children’s Hospital, University of Manitoba, Winnipeg, Manitoba, Canada); Rachel Schrader (Parent Project Muscular Dystrophy), and Kathi Kinnett (Parent Project Muscular Dystrophy). Growth and Puberty Working Group: Co-Chairs - Meilan M. Rutter (Cincinnati Children’s Hospital Medical Center, University of Cincinnati College of Medicine, Cincinnati, Ohio, United States) and Claire L. Wood (Great North Children’s Hospital, University of Newcastle-Upon-Tyne, Newcastle Upon Tyne, United Kingdom); Members - Anne Marie Sbrocchi (Montreal Children’s Hospital, McGill University, Montreal, Québec, Canada), Carol K. L. Lam (The Hospital for Sick Children, University of Toronto, Toronto, Ontario, Canada), Funmbi Babalola (Children’s Hospital, London Health Sciences Centre, The University of Western Ontario, London, Ontario, Canada), Janet L. Crane (Johns Hopkins University, Baltimore, MD, United States), Julia C. Sorbara (The Hospital for Sick Children, University of Toronto, Toronto, ON, Canada), Laura McAdam (Holland Bloorview Kids Rehabilitation Hospital, University of Toronto, Toronto, Ontario, Canada), Robert Benjamin (Duke University Medical Center, Durham, NC, United States), Stefan Nicolau (Nationwide Children’s Hospital, The Ohio State University, Columbus, OH, United States), and Mena Scavina (Parent Project Muscular Dystrophy). Osteoporosis Prevention Working Group: Senior Co-Chairs - Leanne M. Ward (Children’s Hospital of Eastern Ontario, University of Ottawa, Ottawa, Ontario, Canada) and Sze Choong Wong (Royal Hospital for Children, University of Glasgow, Glasgow, United Kingdom); Junior Co-Chairs - Kim Phung (Children’s Hospital of Eastern Ontario, University of Ottawa, Ottawa, Ontario, Canada) and Nat Nasomyont (Cincinnati Children’s Hospital Medical Center, University of Cincinnati College of Medicine, Cincinnati, OH, United States); Members - Anne Marie Sbrocchi (Montreal Children’s Hospital, McGill University, Montreal, Québec, Canada), Cuixia Tian (Cincinnati Children’s Hospital Medical Center, University of Cincinnati, Cincinnati, Ohio, United States), David R. Weber (The Children’s Hospital of Philadelphia, University of Pennsylvania, Philadelphia, PA, United States), Janet L. Crane (Johns Hopkins University, Baltimore, MD, United States), Maria Fernanda Ochoa Molina (Clínica Alemana de Santiago, Santiago, Chile), Meilan M. Rutter (Cincinnati Children’s Hospital Medical Center, University of Cincinnati College of Medicine, Cincinnati, Ohio, United States), Melissa Fiscaletti (Sainte Justine University Hospital, University of Montreal, Montreal, Québec, Canada), Nadia Merchant (University of Texas Southwestern Medical Center, Dallas, TX, United States), Paula R. Clemens (University of Pittsburgh School of Medicine, Pittsburgh, PA, United States), Rana Halloun (Children’s Hospital of Eastern Ontario, Ottawa, Ontario, Canada), Shipra Bansal (University of Arkansas for Medical Sciences, Little Rock, AR, United States), Stefan Nicolau (Nationwide Children’s Hospital, The Ohio State University, Columbus, OH, United States), and Pat Furlong (Parent Project Muscular Dystrophy). Adult Sexuality and Wellness Working Group: Chairs - Susan Apkon (Children’s Hospital Colorado, University of Colorado School of Medicine, Aurora, CO, United States) and Nora E. Renthal (Boston Children’s Hospital and Harvard Medical School, Boston, MA, United States); Members - Amanda M. Appel (Pediatric Rehabilitation Medicine, Children’s Hospital Colorado and University of Colorado School of Medicine, Aurora, CO, United States), Aravind Veerapandiyan (Arkansas Children’s Hospital, University of Arkansas for Medical Sciences, Little Rock, AR, United States), Claire L. Wood (Great North Children’s Hospital, University of Newcastle-Upon-Tyne, Newcastle Upon Tyne, United Kingdom), Gabriela Vargas (Boston Children’s Hospital and Harvard Medical School, Boston, MA, United States), Lindsey E. Bratland (Nationwide Children’s Hospital, Ohio State University, Columbus, OH, United States), Nat Nasomyont (Cincinnati Children’s Hospital, University of Cincinnati College of Medicine, Cincinnati, OH, United States), Natalie Truba (Nationwide Children’s Hospital, Ohio State University, Columbus, OH, United States), Prasanth N. Surampudi (University of California, Davis Medical Center, Sacramento, CA, United States), Janet Hoskin (University of East London, London, United Kingdom), Colin Werth (Parent Project Muscular Dystrophy), and Patrick Moeschen (Parent Project Muscular Dystrophy). Weight Management Working Group: Chairs - Nadia Merchant (University of Texas Southwestern Medical Center, Dallas, TX, United States) and Jaclyn Tamaroff (Vanderbilt University Medical Center, Nashville, TN, United States); Members - Christopher Lewis (Children’s Hospital Colorado, University of Colorado, Colorado, United States), Cuixia Tian (Cincinnati Children’s Hospital Medical Center, University of Cincinnati, Cincinnati, Ohio, United States), Diana X. Bharucha-Goebel (Nationwide Children’s Hospital, Columbus, OH, United States), Philip Zeitler (Children’s Hospital Colorado, University of Colorado Denver School of Medicine, Aurora, CO, United States), Laura McAdam (Holland Bloorview Kids Rehabilitation Hospital, University of Toronto, Toronto, Ontario, Canada), Maria-Elena Lautatzis (Manitoba Children’s Hospital, University of Manitoba, Winnipeg, Manitoba, Canada), Meilan M. Rutter (Cincinnati Children’s Hospital Medical Center, University of Cincinnati College of Medicine, Cincinnati, Ohio, United States), Ryan Farrell (University Hospitals Cleveland Medical Center, Rainbow Babies and Children’s Hospital, Cleveland, OH, USA), Rana Halloun (Children’s Hospital of Eastern Ontario, Ottawa, Ontario, Canada), Sonum Bharill (Johns Hopkins University, Baltimore, MD, United States), Sze Choong Wong (Royal Hospital for Children, University of Glasgow, Glasgow, United Kingdom), and Pat Furlong (Parent Project Muscular Dystrophy).
