# Supplementary material for: A structured approach to address variability in the global management of adrenal insufficiency in Duchenne muscular dystrophy: the 2025 PJ Nicholoff steroid protocol
Source: Front Neurol. 2026 Sep 10;17:1926817. doi: 10.3389/fneur.2026.1926817 (PMC13600995; doi:10.3389/fneur.2026.1926817)
Supplement: Supplementary file 1 [file Table_1.DOCX]

**Supplementary Table 1: Signs and symptoms of glucocorticoid (GC)-induced adrenal insufficiency**

| **Symptoms of GC-induced adrenal insufficiency^1^** | **Signs of GC-induced adrenal insufficiency^1^** |
| --- | --- |
| Anorexia | Hypotension |
| Nausea /vomiting | Hypoglycemia |
| Malaise | Tachycardia or Bradycardia |
| Weakness or fatigue | Fever^2^ |
| Headache |  |
| Abdominal pain |  |
| Myalgia/arthralgia |  |
| Psychiatric symptoms (confusion, delirium, disorientation) |  |
| Loss of motor skills (out of keeping with natural progression) |  |

^1^ Symptoms of GC withdrawal may mimic symptoms of adrenal insufficiency. Note that not all symptoms and/or signs are always present. Hypotension and hypoglycemia typically do not occur with GC withdrawal.

^2^ Fever is an uncommon sign of adrenal insufficiency but may occur in isolation in rare instances. The source of fever should always be investigated and treated, as appropriate.
