# Supplementary material for: A structured approach to address variability in the global management of adrenal insufficiency in Duchenne muscular dystrophy: the 2025 PJ Nicholoff steroid protocol
Source: Front Neurol. 2026 Sep 10;17:1926817. doi: 10.3389/fneur.2026.1926817 (PMC13600995; doi:10.3389/fneur.2026.1926817)
Supplement: Supplementary file 2 [file Table_2.DOCX]

**Supplementary Table 2: Maintenance doses and stress dosing considerations for glucocorticoids in Duchenne muscular dystrophy**

| **Gluccocorticoid** | **Used for DMD Treatment** | **Maintenance dose^1^** | **Half-life (hours)^2^** | **Glucocorticoid receptor activity** | **Mineralocorticoid receptor activity** | **Appropriate for stress coverage^3^** |
| --- | --- | --- | --- | --- | --- | --- |
| **Hydrocortisone** | NO | 8-10 mg/m^2^/day | 3 | Agonist | Agonist | YES |
| **Prednisone** | YES | 2-3 mg/m^2^/day | 6.2 | Agonist | Agonist | YES |
| **Prednisolone** | YES | 2-3 mg/m^2^/day | 6.2 | Agonist | Agonist | YES |
| **Deflazacort** | YES | 2.6 mg/m^2^/day^4^ | 1.1 to 1.9 | Agonist | Weak agonist | UNKNOWN^5^ |
| **Vamorolone** | YES | Unknown | 2 | Agonist | Antagonist | NO |

^1^ Expressed as mg/m^2^ of body surface area (BSA)/day.

^2^ Clinically a drug is considered to be eliminated from the body after 4-5 half-lives if it is discontinued

^3^ See Table 3 for stress dosing details

^4^ Estimates based on historically accepted conversion factors and should be considered as a guide only. There is insufficient data to inform optimal dosing of deflazacort for maintenance replacement

^5^ Insufficient data to inform stress dose or frequency. Use with caution

Abbreviations: DMD, Duchenne Muscular Dystrophy
