# Supplementary material for: A structured approach to address variability in the global management of adrenal insufficiency in Duchenne muscular dystrophy: the 2025 PJ Nicholoff steroid protocol
Source: Front Neurol. 2026 Sep 10;17:1926817. doi: 10.3389/fneur.2026.1926817 (PMC13600995; doi:10.3389/fneur.2026.1926817)
Supplement: Supplementary file 3 [file Table_3.DOCX]

**Supplementary Table 3: Continuous IV hydrocortisone infusion rates based on measured weight as an alternative to intermittent doses for severe stress**

| **Weight** | **Total dose in 24 hours** | **Hydrocortisone infusion rate^1^** |
| --- | --- | --- |
| ≤ 10kg | 24 mg | 1 mL/hour |
| 10.1 to 20kg | 48 mg | 2 mL/hour |
| 20.1 to 40kg | 96 mg | 4 mL/hour |
| 40.1 to 70kg | 144 mg | 6 mL/hour |
| Over 70kg | 192 mg | 8 mL/hour |

**^1^** Hydrocortisone infusion for IV use prepared as 50 mg hydrocortisone in 50 mL 0.9% sodium chloride (1 mg/mL)

Adapted from *Mushtaq T, et al. Archives of disease in childhood. 2023;108(11):871-8*

Abbreviations: IM, intramuscular; IV, intravenous
