# Supplementary material for: A structured approach to address variability in the global management of adrenal insufficiency in Duchenne muscular dystrophy: the 2025 PJ Nicholoff steroid protocol
Source: Front Neurol. 2026 Sep 10;17:1926817. doi: 10.3389/fneur.2026.1926817 (PMC13600995; doi:10.3389/fneur.2026.1926817)
Supplement: Supplementary file 4 [file Table_4.DOCX]

**Supplementary Table 4: Example approaches to transitioning individuals from one glucocorticoid (GC) or GC regimen to another^1,2^**

| **GC Transition Scenario** | **Proposed management** |
| --- | --- |
| **Daily prednisone/prednisolone or deflazacort to vamorolone dose of 6 mg/kg/day or max dose (recommended approach)** | - No tapering needed; can switch directly to vamorolone 6 mg/kg/day or max dose |
| **Daily prednisone/prednisolone or deflazacort to vamorolone dose < 6 mg/kg/day (or < max dose^3^** | - In addition to desired vamorolone dose, initiate hydrocortisone, prednisone/prednisolone at a maintenance dose **(See Table 2, Step 1)** - Suggest continuing for 2-4 weeks after transition to facilitate smooth change from one to the other, and then stop - If symptoms of GC withdrawal develop, initiate stress dosing as per stress steroid plan followed by a gradual taper back to maintenance dose |
| **Vamorolone to prednisone/prednisolone 0.75 mg/kg/day or deflazacort 0.9 mg/kg/day (or max dose per prescribing clinician, recommended approach)** | - No tapering needed; can switch directly to prednisone/prednisolone 0.75 mg/kg/day or deflazacort 0.9 mg/kg/day |
| **Vamorolone to prednisone/prednisolone doses < 0.75 mg/kg/day or deflazacort doses < 0.9 mg/kg/day** | - Switch directly to desired dose and no tapering needed **only if** desired prednisone/prednisolone or deflazacort treatment dose exceeds the following supraphysiologic (triple maintenance) doses^4^:   **Prednisone/Prednisolone:**  **10-25 kg: 5 mg daily**  **26-50 kg: 10 mg daily**  **51-90 kg: 15 mg daily**  **Deflazacort:**  **10-25 kg: 6 mg daily**  **26-50 kg: 12 mg daily**   51-90 **g: 18 mg daily** - If desired prednisone/prednisolone or deflazacort dose is less than above doses, switch to dose of prednisone/prednisolone or deflazacort as above and then taper by 2.5-5 mg/day (prednisone/prednisolone) or 3-6 mg/day (deflazacort) every 1-2 weeks until desired dose is reached |
| **Daily prednisone/prednisolone, deflazacort, or vamorolone to twice-weekly prednisone, prednisolone, or deflazacort doses** | - Such transitions should be done over 6-8 weeks with gradual dose reduction on “off days” (no GC) in parallel with equivalent dose increases on “treatment days” until twice-weekly day dosing is reached. If symptoms of GC withdrawal or adrenal insufficiency develop on “off days” restart previous dosing on “off days” and perform a more gradual reduction of the dose on “off days” in parallel with an increase in the dose on “treatment days”. - Consult endocrinology, if available, and consider testing HPA axis after 1-6 months on “off days” (**Table 2, Step 3**), to determine if ongoing stress steroid plan is needed |
| **Daily prednisone/prednisolone, deflazacort, or vamorolone to intermittent prednisone/prednisolone or deflazacort doses (ie. 10 days on 10 days off or any other intermittent regimen that is not twice-weekly)** | - For “OFF days” (no GCs), start maintenance prednisone/prednisolone, or hydrocortisone **(see Table 2, Step 1)**   - Suggest continuing for 4 weeks and then stopping. If symptoms of GC withdrawal develop, restart maintenance dosing and consider a longer duration of treatment or a taper off maintenance doses - For “ON days” (with GCs):   - If intermittent prednisone/prednisolone or deflazacort treatment day dose exceeds supraphysiologic (triple maintenance) dose (**see doses above)** then switch directly to desired intermittent prednisone/prednisolone or deflazacort “treatment day” dose   - If desired intermittent prednisone/prednisolone or deflazacort treatment day dose is less than maintenance, switch to supraphysiologic (triple maintenance) dose of prednisone/prednisolone or deflazacort **(see doses above**) on treatment days and then taper by 2.5-5 mg/day (prednisone/prednisolone) or 3-6 mg/day (deflazacort) every 1-2 weeks until intermittent “treatment day” dose reached - Consult endocrinology, if available, and consider testing HPA axis after 1-6 months (**Table 2, Step 3**), to determine if routine ongoing stress steroid plan is needed |

^1^ Many of these regimens are used off label in clinical practice or are inferior (e.g.10 days on 10 days off prednisone) for motor function, and maximum doses may vary according to country-specific regulatory approvals and prescribing clinician practices

^2^ Ensure first day of new GC dosing directly follows last day of previous GC such that no doses are missed

^3^ Maximum vamorolone dose: 300 mg in the USA, 240 mg in the EU, UK, China, and Canada

^4^ Doses are rounded to correspond with available medication formulations

Abbreviations: GC, glucocorticoid; max, maximum
